# Rapidly Progressive Constrictive Pericarditis Occurring 10 Days After Purulent Pericarditis

**DOI:** 10.7759/cureus.114076

**Published:** 2026-08-06

**Authors:** Yuki Hayashi, Koichiro Hori, Naoki Eguchi, Riku Arai, Masashi Tanaka

**Affiliations:** 1 Department of Cardiovascular Surgery, Nihon University School of Medicine, Tokyo, JPN; 2 Division of Cardiology, Department of Medicine, Nihon University School of Medicine, Tokyo, JPN

**Keywords:** acute constrictive pericarditis, constrictive pericarditis, pericardiectomy, purulent pericarditis, suppurative pericarditis

## Abstract

Constrictive pericarditis (CP) typically develops after a chronic course of infection or other etiologies. We report a rare case of acute CP that manifested rapidly following purulent pericarditis (PP). A 63-year-old woman with a history of hematologic disease presented with dyspnea and fever. Pericardial effusion with signs of infection was noted, and emergency pericardial drainage was performed because of hemodynamic instability. *Haemophilus influenzae* was detected in the pericardial fluid; consequently, antibiotic therapy was initiated. However, the patient’s condition deteriorated. Transthoracic echocardiography revealed diastolic dysfunction, leading to the diagnosis of acute CP on hospital day 10 after pericardiocentesis. Given the progressive hemodynamic compromise and treatment failure, open pericardiectomy with pericardial lavage was performed, resulting in rapid improvement in hemodynamics. The patient was discharged ambulatory on postoperative day 28. Although rare, PP can rapidly progress to CP; therefore, early surgical intervention should be considered in cases that do not respond to antibiotic therapy or pericardial drainage.

## Introduction

Constrictive pericarditis (CP) is defined as a form of pericarditis in which the pericardium, which forms a shell around the heart that lacks elasticity, impedes the filling of the heart during diastole. CP causes diastolic dysfunction and can lead to heart failure, with an annual incidence reported at approximately 0.1%, according to a systematic review of contractile pericarditis in patients from Asia, Africa, Europe, and North America [1]. CP is a rare complication associated with acute pericarditis. In developing countries, tuberculous pericarditis accounts for approximately 50% of the cases, whereas in developed countries, idiopathic and viral causes account for approximately 40%, and purulent pericarditis (PP) accounts for approximately 5% [2]. Most cases of CP manifest gradually over several months [2]. In contrast, PP has become rare since the advent of antibiotics, but it is associated with a high mortality rate even with appropriate treatment and is highly likely to progress to CP [3]. Here, we report a rare case of rapidly progressive CP developing within two weeks of the onset of PP, which was successfully and timely treated with surgical intervention.

## Case presentation

A 63-year-old woman with a history of angioimmunoblastic T-cell lymphoma, in remission for six years following allogeneic hematopoietic stem cell transplantation, presented to our hospital with a history of fever and cough for two weeks. Lymphoma was well controlled without any oral medication, and she was not immunocompromised. Her dyspnea had progressively worsened prior to admission. Upon arrival at the clinic, the patient’s blood pressure was 71/50 mmHg, pulse rate was 130 bpm, and SpO₂ was 90%. Electrocardiography revealed diffuse ST-segment elevation. Laboratory tests indicated leukocytosis with neutrophil predominance and elevated C-reactive protein levels (Table 1).

**Table 1:** Blood test results at the time of visit BUN: Blood urea nitrogen; T-bil: total bilirubin; APTT: activated partial thromboplastin time; AST: aspartate aminotransferase; ALT: alanine transaminase; PT: prothrombin time

Blood test	values	Reference range
WBC	42800/μL	4000-8000/μL
neutrophil	94%	45-75%
CRP	32.44mg/dL	<0.3mg/dL
procalctionin	0.78ng/mL	<0.05ng/mL
lactate	4mg/dL	0.5-2.0mg/dL
creatinine	1.34mg/dL	0.47-0.79mg/dL
BUN	17.2mg/dL	8-20mg/dL
NT-proBNP	4830pg/mL	<125pg/mL
Troponin I	0.01ng/mL	<0.01ng/mL
T bil	0.65mg/dL	0.2-1.2mg/dL
AST	23U/L	<30U/L
ALT	18U/L	<30U/L
PT	69%	70-100%
PT-INR	1.26	0.85-1.15
APTT	27.6 sec	20-40sec
D-dimer	4.1μg/mL	<1.0μg/mL

**Figure 1:**
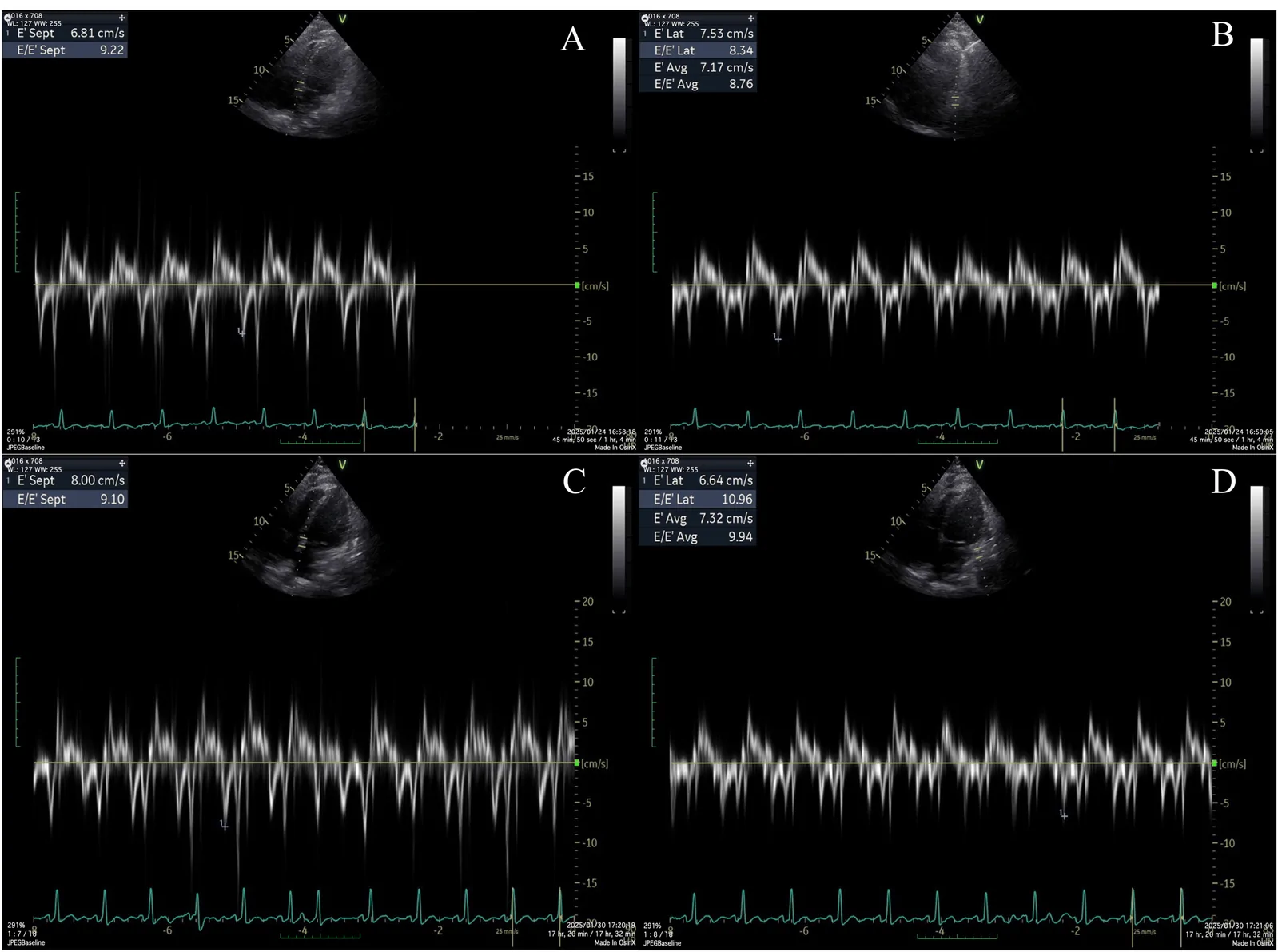
Transthoracic echocardiography on admission and on day 12 On admission (A and B), mitral annular velocities (e′) were 6.81 cm/s at the septal side and 7.53 cm/s at the lateral side. On day 12 (C and D), these values changed to 8.00 cm/s at the septal side and 6.64 cm/s at the lateral side, demonstrating that septal e′ > lateral e′ and indicating constrictive physiology.

Transthoracic echocardiography revealed a large pericardial effusion, which raised the suspicion of PP. Subsequently, emergency pericardial drainage was performed because of hypotension and signs of cardiac tamponade. The drained fluid was turbid and appeared yellowish-brown, consistent with PP. Haemophilus influenzae was detected in the pericardial fluid culture and blood culture, and intravenous meropenem (500 mg every 8 h) was promptly initiated. However, despite 12 days of antibiotic treatment by meropenem, the patient developed septic shock and required catecholamine support. Subsequent transthoracic echocardiography revealed no evidence of diastolic dysfunction. On hospital day 1, her respiratory condition deteriorated because of worsening pneumonia and sepsis-associated acute respiratory distress syndrome, necessitating endotracheal intubation and mechanical ventilation. Despite intensive antibiotic therapy, systemic inflammation persisted. A follow-up echocardiogram performed on hospital day 12 revealed septal bounce (Video 1), increased mitral annular velocity (E′), and medial E′ greater than lateral E′, suggesting newly developed diastolic dysfunction (Figure 1).

**Video 1:** Transthoracic echocardiography on day 12 of hospitalization Transthoracic echocardiography revealed a newly developed septal bounce.

**Figure 2:**
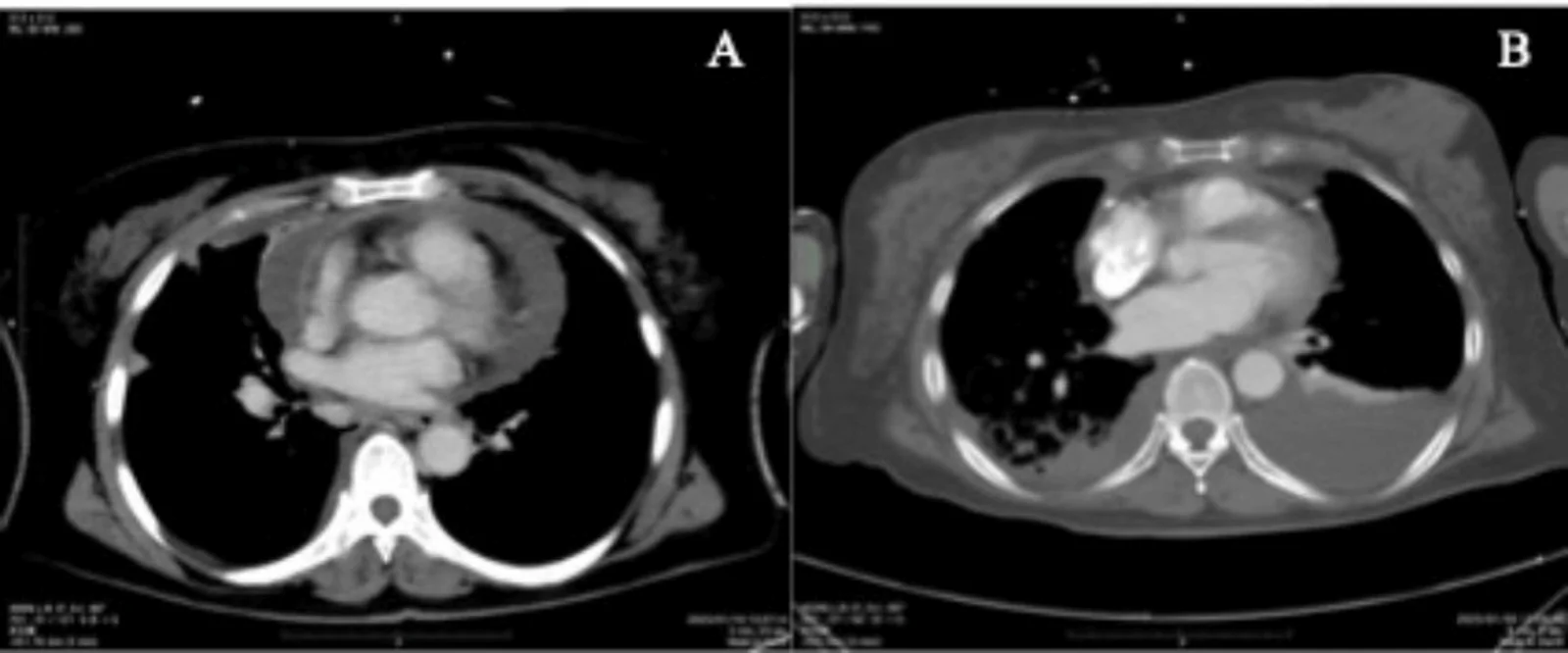
CT scan, on admission and on day 12 of hospitalization Pericardial effusion was noted on admission (A), but the volume decreased after drainage via pericardiocentesis (B). However, progressive diastolic dysfunction was observed, raising suspicion of constrictive pericarditis. Nevertheless, no obvious pericardial calcification, significant pericardial thickening (pericardium 4 mm), or signs of pericardial inflammation were observed (B).

**Figure 3:**
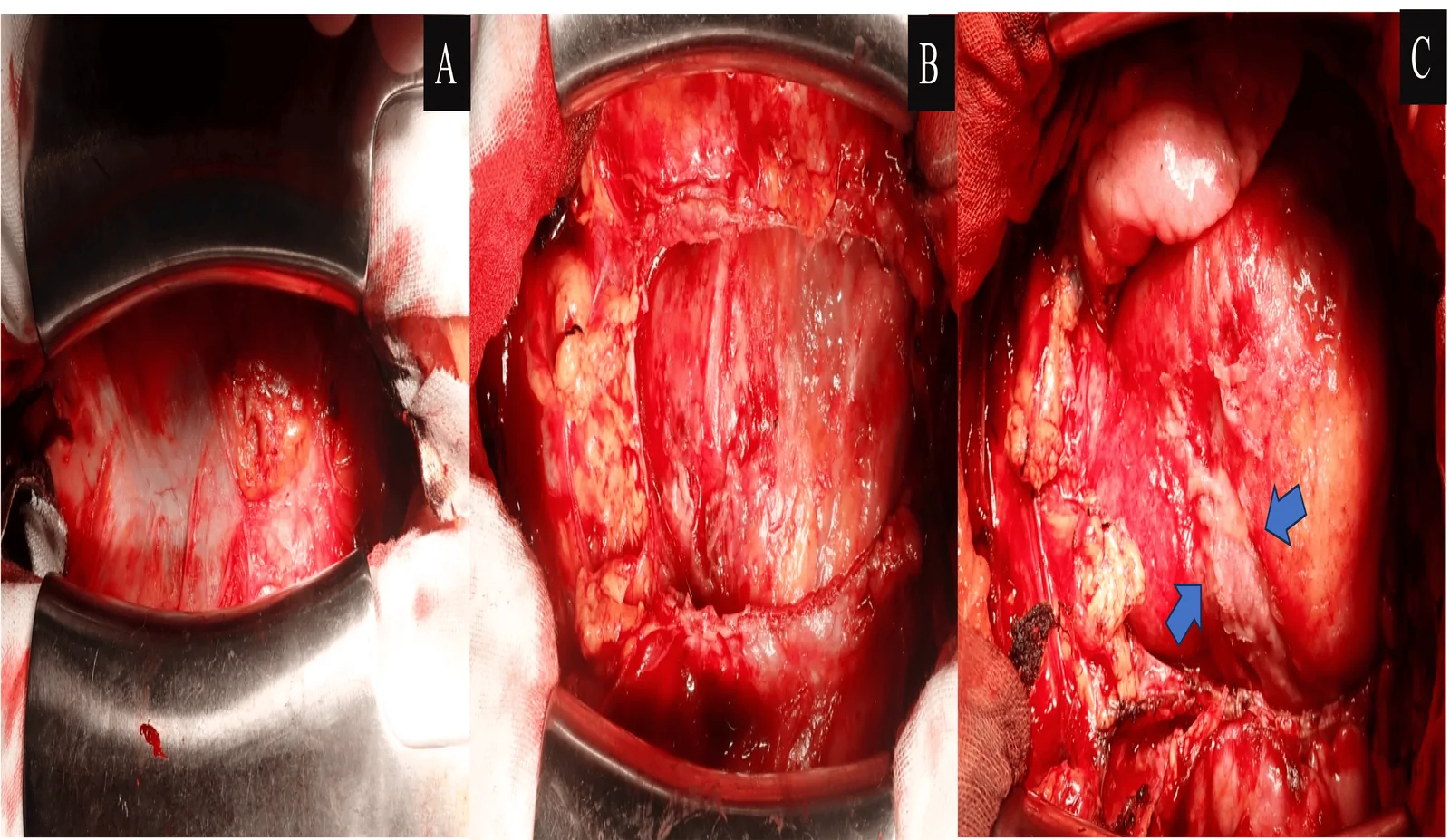
Intraoperative findings during median sternotomy The pericardium appeared rigid with a loss of elasticity (A). The anterior pericardium was carefully released (B), followed by extensive pericardial dissection and resection extending up to the bilateral phrenic nerves and the inferior surface of the heart (C). Fibrinous adhesions were observed on the anterior surface of the heart (arrow in panel C).

These findings were consistent with early progression to CP. Computed tomography did not demonstrate significant pericardial thickening (Figure 2), and right heart catheterization did not show the typical dip-and-plateau pattern. Based on these findings and multidisciplinary exchanges, the patient was diagnosed with rapidly progressive constrictive pericarditis. Given the persistent hemodynamic instability and medical therapy failure, surgery was deemed necessary. Median sternotomy was performed under general anesthesia. Intraoperatively, the pericardium was thickened, stiff, with adhesions in the epicardium, and a small amount of cloudy yellow fluid. Extensive pericardiectomy was performed on the anterior, lateral, and inferior surfaces of the heart. Fibrin deposition was observed on the epicardial surface, suggesting active inflammation. However, no fibrosis was noted, indicating an acute inflammatory response rather than chronic fibrotic constriction (Figure 3).

Cardiac contractility improved immediately after pericardial resection, and the diastolic dysfunction resolved (Video 2).

**Video 2:** Intraoperative findings after pericardiectomy The video demonstrates improved cardiac motion without evidence of epicardial adhesion or diastolic dysfunction.

The operative field was irrigated with saline, and the chest was closed. Hemodynamic parameters improved after pericardiectomy, with right ventricular systolic, diastolic, and mean pressures decreasing from 35/14/23 mmHg preoperatively to 29/9/17 mmHg postoperatively. Although epicardial intervention was considered, the intraoperative findings suggested no residual diastolic dysfunction; therefore, pericardiectomy alone was deemed effective. Postoperative echocardiography confirmed resolution of the restrictive physiology. Continuous pericardial irrigation was performed for four days via a pericardial drain, which was discontinued after confirming negative bacterial cultures. The patient’s clinical condition steadily improved, and she was discharged on postoperative day 28.

## Discussion

This case highlights the rare and rapid progression of PP to CP within a short period. Most cases progress over weeks to months [4,5], whereas our patient developed constrictive physiology within approximately 10 days. Acute CP is characterized by transient constrictive physiology associated with active inflammation, which may be reversible in some cases [6]. Previous studies have shown that the risk of developing CP after acute pericarditis is low overall but significantly higher in cases associated with bacterial infection [2,7]. Although rare in the antibiotic era, PP is associated with high mortality and severe complications, including cardiac tamponade and hemodynamic instability. According to a large-scale, long-term follow-up study by Imazio et al. [2], the risk of developing chronic pericarditis following an initial episode of acute pericarditis is approximately 1.8%, suggesting low occurrence. However, the study also demonstrated that acute pericarditis resulting from bacterial pericarditis carries a high risk, with approximately 33% of cases progressing to chronic pericarditis [2,8]. Furthermore, the study also states that persistent symptoms, massive accumulation of pericardial fluid, and failure of empirical anti-inflammatory therapy are critical risk factors for CP. In the present case, early echocardiographic detection of subtle diastolic dysfunction was critical for accurate diagnosis. Notably, typical findings, such as pericardial thickening on computed tomography or dip-and-plateau patterns on catheterization, were absent, which made early diagnosis challenging. Prompt surgical intervention resulted in a favorable outcome. Intraoperative findings suggested that the inflammation was limited to the pericardium without epicardial involvement, allowing for successful treatment with pericardiectomy alone. Although some cases of acute CP may resolve with anti-inflammatory therapy, surgical intervention was necessary in this case because of progressive hemodynamic compromise. These findings suggest that early surgical intervention should be considered in patients with PP who develop signs of hemodynamic compromise even in the absence of classical diagnostic findings. This case underscores that echocardiographic findings may precede classical imaging or hemodynamic features, highlighting the importance of early clinical suspicion.

## Conclusions

We present a rare case of rapidly progressive CP following PP that was successfully treated with early surgical intervention. Although antibiotics and drainage are usually effective in treating suppurative pericarditis, clinicians should be aware that, as in this case, restrictive pericarditis can develop within a short period after onset. Early recognition and timely surgical intervention are essential for improving clinical outcomes, particularly in patients showing rapid clinical deterioration.
